# Variation in Implementing Dementia-Friendly Community Initiatives: Advancing Theory for Social Change

**DOI:** 10.3390/geriatrics8020045

**Published:** 2023-04-21

**Authors:** Clara J. Scher, Emily A. Greenfield

**Affiliations:** Rutgers School of Social Work, 120 Albany Street, New Brunswick, NJ 08901, USA

**Keywords:** dementia-friendly communities, community capacity, qualitative research, implementation

## Abstract

Dementia-friendly communities (DFC) have emerged as a global movement to make communities more supportive and inclusive of people living with dementia (PLWD) and their care partners. This study contributes to a nascent body of research on DFC initiatives by building theory on their local implementation. Based on an analysis of data from semi-structured interviews with 23 leaders of initiatives in Massachusetts (United States), we aimed to identify key dimensions of variation in the implementation of DFC initiatives. We found that all initiatives engaged in a common set of activities, such as the facilitation of training about dementia and improving services for PLWD. Although initiatives mostly engaged in these activities in ways that targeted the community at large, in some instances, they concentrated their efforts on enhancing the dementia-friendliness of their own organizations. We describe ways in which financial, social, and human capital operate as key factors that influence the initiatives’ primary focus (i.e., the community at large or their own organization). Our findings suggest the importance of helping DFC initiative leaders more explicitly specify the focal ecological level of their efforts throughout the trajectory of their work, especially in the context of resource considerations. Results also indicate ways in which DFC initiative efforts at one systems level can support those at other levels over time.

## 1. Introduction

Dementia-friendly communities (DFCs) embody a global movement to make localities, systems, environments, and social institutions more supportive of people living with dementia (PLWD) and their care partners [1]. Work on DFCs began in Japan as early as 2004, with a nationwide campaign to better understand dementia and build supportive community networks, which inspired the growth of the movement worldwide [1]. In particular, the United Kingdom has emerged as a champion of the dementia-friendly movement; beginning in 2012, the Prime Minister’s Challenge on Dementia called for the development of DFCs [2,3], and a nationwide recognition program led by the Alzheimer’s Society guides and monitors local communities’ efforts [4,5].

In the United States, the DFC movement is relatively new and evolving. Uptake emerged most formally in 2013 with Minnesota’s Act on Alzheimer’s, a statewide initiative seeking to prepare communities to support PLWD and their care partners [6]. The pioneering work of Act on Alzheimer’s inspired the development of Dementia-Friendly America (DFA) [7]. DFA is a national initiative led by USAging, a national nonprofit that represents Area Agencies on Aging (e.g., the federally mandated structure for regional offices to help older adults and people with disabilities age well in their communities) [8]. DFA, with the support of organizations from the public and private sectors [9], encourages the development of DFCs across the United States [1,7]. Although DFC work in the United States generally has been organized at the state and local levels, there is a growing number of federal initiatives related to DFCs. Examples include the United States Centers for Disease Control and Prevention’s (CDC) Alzheimer’s Disease and Healthy Brain Initiative [10] and the Administration for Community Living’s (ACL) Alzheimer’s Disease Programs Initiative [11]. Moreover, discourse on *age*-friendly communities continues to emphasize the value of incorporating DFC-related goals and activities [12,13].

Despite the emergence of DFC efforts in the United States, empirically based understanding of how the work is happening—especially within local communities—is in its nascence. Advancing knowledge in this area is important to support the expansion, sustainability, and impact of the movement. For example, insights on DFC practice grounded in the perspectives of community leaders can help to inform existing and future efforts to support local communities in launching and maintaining DFC efforts. It also is important for guiding the design of future research and evaluation on DFC initiatives by delineating benchmarks for implementation that would lead to desired outcomes. Toward this aim, our study uses qualitative methods to identify dimensions of variation across local leaders’ narratives concerning the programmatic emphases of their DFC work and to delineate contexts that facilitate key differences in DFC initiative implementation.

### 1.1. Empirical Background

Alzheimer’s Disease International—an organization that connects more than 100 dementia-focused associations across the world [14]—defines DFCs as “a place or culture in which people with dementia and their care partners are empowered, supported and included in society, understand their rights, and recognize their full potential” [15]. Guided by prior discourse [15,16,17,18,19,20,21,22], we conceptualize DFC *initiatives* as systematic efforts to make communities more inclusive of persons living with a diversity of cognitive abilities as they age; DFC initiatives further strive to make communities more supportive of PLWD and their care partners by facilitating the collaboration and contributions of community leaders across sectors, including PLWD.

The literature to date has focused largely on describing characteristics of DFC initiatives through case illustrations [23,24,25,26] and reflections on practice [20,21,27]. In addition, several review articles have summarized various aspects of DFC initiatives, such as general characteristics and strategies of DFC initiatives (e.g., multisectoral collaboration, involvement of PLWD) [16,17,19], how DFC initiatives can promote social inclusion (e.g., inclusive environmental design and public education to reduce dementia-related stigma) [28], and dementia-friendly efforts to improve the built environment (e.g., easily accessible social and retail destinations, meaningful landmarks) [29].

Empirical research on DFC initiatives as a programmatic set of activities has largely come from the United Kingdom. In England, the DEMCOM study conducted a national evaluation of DFC initiatives to understand the ways they support PLWD and their care partners, and also to explore DFC initiative sustainability [30]. This work has resulted in the development of a DFC evaluation framework (e.g., thematic areas that should be considered when evaluating DFC initiatives), theory of change (e.g., how initiatives can make a difference and what outcomes they can achieve at what stage), and matrix for assessing initiative maturity (e.g., criteria for characterizing stages of DFC initiative development) [31]. The findings revealed that policy supports, a formal DFC initiative recognition process, and strong local collaborations are important in the sustainability of DFC work [30].

There is only one study in the United States, to our knowledge, that has utilized formal research methods to investigate DFC initiatives. Sun and colleagues (2022) conducted semi-structured interviews with 17 stakeholders involved in DFC initiatives in the United States and China to understand challenges and strategies during the COVID-19 pandemic [18]. Challenges encompassed difficulties involving PLWD due to social distancing protocols and limited policy supports for DFC initiative work. Strategies included drawing on partnerships with local government and foundations to fund the initiatives, as well as utilizing Zoom technology to engage PLWD and their care partners [18]. The authors recommend future directions for DFC initiative practices, including advocating for public funds to support DFC work and hiring PLWD to lead DFC initiatives.

### 1.2. Focus of the Current Study

Our study aims to contribute to the emerging international literature on DFC initiative development by exploring how local leaders conceptualize, describe, and organize their initiative activities. More specifically, we use qualitative methods to develop theory that explicates dimensional differences across DFC initiative implementation. Our study is premised on the assumption that initiatives vary from one community to the next in theoretically meaningful ways. Although state and national leaders have promulgated frameworks to guide communities’ efforts [7,32], there is anecdotal evidence to suggest that “if you have seen one dementia-friendly community, you have seen one dementia-friendly community” [31,33]. As Buckner and colleagues (2022) explain, because DFC initiatives are to be rooted within their communities, it is expected that they are designed and implemented in community-specific ways [31]. Moreover, guidelines for joining networks such as DFA are flexible, allowing for a range of types and degrees of work to be included.

We also aim to explore what contextual factors influence variation in implementation. Prior scholarship has begun to identify conditions that contribute to the start-up and maintenance of DFC initiatives, such as funding sources, multisectoral partnerships, and leadership [18,19]. Our study aims to advance this area of knowledge by exploring how organizational and community contexts potentially lead to different approaches for the implementation of DFC initiatives.

## 2. Materials and Methods

### 2.1. Study Setting

Our study was conducted in Massachusetts (MA), a state located in the northeastern region of the United States. MA has uniquely organized a statewide dementia-friendly network involving leadership across private nonprofit organizations and state government agencies [34]. MA is further unique given state policy that requires all municipalities to have a local Council on Aging (COA). In MA, COAs mostly operate as municipal older adult activity centers (with or without their own dedicated buildings), serving as a hub for community-based services as well as providing opportunities for social connection and engagement [35]. In many cases, COAs have served as the local champion for DFC initiative implementation efforts throughout MA.

Regarding MA’s statewide initiative, Dementia Friendly Massachusetts (DFM) began in 2016 with private philanthropic support. DFM aims to spur the development of local DFC initiatives through a state-level recognition, advocacy, and technical assistance program. DFM also facilitates and sustains a multisectoral statewide leadership team to guide this work at the state, regional, and municipal levels. Cities and towns can join the DFM network by signing a pledge, with commitment from their local governments; DFM calls for its members to promote the inclusion and empowerment of PLWD and their care partners through creating innovative programs and enhancing local services [36]. The Massachusetts Councils on Aging (MCOA), a nonprofit organization, serves as the administrative lead for DFM [32].

### 2.2. Sample

Representatives from the 24 communities that had signed the DFM pledge at the municipal level by March of 2021 were invited to participate in a Zoom-based qualitative interview with the research team at that time. All but four communities were interviewed (non-participating communities varied in their population size and median household income, and some were experiencing transitions in their DFC initiative leadership during the period of our interview request). In addition, one community we interviewed was not included in the final sample because our discussion with the participant in the context of the interview revealed that the DFC initiative was not active. As such, for the purposes of this study, the sample included 19 communities.

The 19 interviews were conducted with a total of 23 individuals, as some of the interviews were conducted with more than one leader from a respective initiative. All participants identified as non-Hispanic White (100%), and the majority of participants were women (91%) and COA directors or outreach coordinators (70%). Most of the initiatives’ administrative auspice was the municipal COA (*n* = 13); however, several of the initiatives were led by other types of organizations, including an assisted living facility (*n* = 1), home care organization (*n* = 1), regional offices on aging (*n* = 2), and grassroots community residents (*n* = 2). All initiatives were implemented at the local municipal level, with the exception of one regionally organized initiative. Table 1 provides an overview of the demographic characteristics of the communities included in our sample, indicating their wide range in population size, percentage of population ages 65 and older, racial/ethnic composition, and socioeconomic status.

### 2.3. Data Collection

The authors conducted the semi-structured interviews via Zoom, which were 90 to 120 min in length, in the spring through summer of 2021. The semi-structured interview guide consisted of open-ended questions customized for each community based on the research team’s background preparation (e.g., review of initiative websites and press releases). Questions addressed how the DFC initiative began, as well as how it was structured and implemented over time. See Supplementary Appendix S1 for an overview of the interview guide as well as the rationale for key sections.

The study received approval from the Institutional Review Board at Rutgers, The State University of New Jersey prior to data collection. Participants signed an online informed consent that included information about their rights as participants and assurances regarding the confidentiality of their interview data.

### 2.4. Data Analysis

All interviews were audio-recorded, transcribed, and imported into NVivo Version 12 for analysis. We used techniques from grounded theory—including memo writing, iterative phases of coding, and matrices—to derive insights on the various approaches to DFC initiative implementation. We used peer debriefing and memo writing throughout all phases of the analysis as techniques to enhance credibility and rigor in qualitative research [37]. For example, after each interview, we completed a structured memo that summarized the goals of the initiative’s work, the structure of their initiative, the focus on partnership development, the experience of statewide support, hopes for the future, as well as overall challenges and opportunities and our own emerging ideas regarding analytic directions and patterns. During the coding of the interview transcripts, we maintained a memo detailing how the codes evolved across our iterative engagement with the text.

Supplementary Table S1 displays the codes and subcodes that emerged across three iterative phases of coding. In summary, phase one involved open coding based on sensitizing concepts (e.g., involvement of PLWD, action teams) derived from our analytic memos and the literature [38,39,40,41]. Open coding involves “identifying distinct concepts and themes for categorization” and “creating initial broad thematic domains for data assemblage” [42]. In phase two, CS sub-coded the excerpts under each of the initial thematic categories and moved subcodes and excerpts originally interpreted under a preliminary theme to a different conceptual category as appropriate. In phase 3, both authors collaboratively read through the codes from phase 2 and conducted axial coding to “aggregate the most closely related or overlapping open codes“ and “categorize the codes with the goal of creating distinct thematic categories in preparation for selective coding” [42].

The fourth stage of analysis involved selective coding or “selecting and integrating categories of organized data…at a higher level of abstraction” [42]. This stage involved reflecting on the codes, writing additional memos on the trajectory of each initiative over time, and developing a matrix. The matrix was organized such that each of the 19 initiatives was displayed as a row, and each of the columns represented the codes and subcodes from the third and final phase. This matrix facilitated the constant comparative method to determine different approaches to DFC initiative implementation, as well as to construct a theory on organizational and community contexts that influence differences. The constant comparative method was used “with the goal of discerning conceptual similarities, refining the discriminative power of categories, and discovering patterns” [43]. This process resulted in the development of the thematic findings, as presented below. The authors then discussed the selection of initiatives to present as case illustrations of the themes, prioritizing cases that differed from each other in theoretically important ways.

## 3. Results

Across all interviews, DFC initiative leaders described their work as striving to make their local environments more inclusive of PLWD and caregivers. Collectively, they identified a common set of activities toward this goal, including the facilitation of training and community outreach events about aging with dementia (e.g., workshops at the local library, training for emergency responders), the development of new services (e.g., memory cafés), and advocating for improvements to existing community resources for PLWD (e.g., discussing with local officials design considerations for outdoor spaces). Despite these similarities, our analysis indicated a key difference across the initiatives: the degree to which the participants described going about these activities in ways that would make the community at large more dementia-friendly in contrast to engaging in these activities in ways to enhance the dementia-friendliness of their own organizations. 

In most cases, there was strong evidence from the interviews that the initiatives—especially at the beginning of their development—were targeted to address the community across their entire locality (i.e., encompassing residents, municipal departments, local businesses, other community-based organizations, etc.). These activities were often described as involving intentional partnerships across several sectors of the community, including public, private, civic, and academic organizations. Initiatives with this community-wide focus were especially likely to develop a multisectoral action team comprising leaders and community residents that actively collaborated on DFC initiative work. 

In contrast, several initiatives were focused on enhancing the dementia-friendliness of their own organizations from the start of their work, and others became more focused on their own organizations as their work evolved over time. In these cases, the initiatives primarily targeted the auspice organization’s staff, volunteers, clients, and facilities with their dementia-friendly efforts. For example, these initiatives conducted training on dementia for their own organizational staff and volunteers. They also largely focused on enhancing the dementia-inclusivity of their service offerings, most commonly adding dementia-centered programming. Initiatives focused on their lead organizations were less likely to have a formal dementia-friendly action team and did not often partner with other organizations on dementia-friendly work.

Many initiatives drew on both approaches simultaneously, especially over the long-term trajectory of their work. For example, some initiatives reported conducting dementia-friendly training with outside community groups while also developing supportive programs for clients of their own organization living with dementia. However, in some cases, as illustrated through the case studies below, we found the initiatives to be more consistently focused on their own organization alone. 

### 3.1. Contexts Influencing Different Approaches to Implementation of DFC Initiatives

Our analysis indicated three thematic categories that describe key contexts influencing the above-described difference in the DFC initiative implementation approach (i.e., focus on the community at larger in contrast to their own organization): financial, social, and human capital.

Financial Capital. Some initiatives received outside funding to support their DFC efforts from sources such as local foundations, regional aging services organizations, and municipal government. Several initiatives that had outside funding were able to hire staff members to coordinate the initiative, pay for dementia-friendly programming, and conduct community-wide assessments. As a result, initiatives with dedicated funding were often able to focus their efforts on the community at large because having financial capital resulted in additional human and social capital to do so. For example, one initiative received foundation funding to hire a paid coordinator for 20 h a week to oversee DFC initiative operations. The leader noted how this unique funding opportunity was integral to the success of the community-wide initiative because without a full-time coordinator, who is “absolutely invaluable and responsible for moving everything ahead…a dementia-friendly initiative would not have been possible”.

Initiatives without outside funding did not hire paid DFC initiative staff, often did not engage in partnerships with outside organizations, and were overall more focused on their own organizations. There were also some initiatives that had outside funding at the outset of their efforts, which allowed for community-level work; however, when funding was no longer available, the initiatives transitioned to a more organization-focused approach. For example, one initiative that was supported by a grant from the regional aging services access point developed a multisectoral action team and created programs that targeted the community at large (e.g., dementia-friendly training for first responders). However, when the grant ended, the action team no longer met, and the DFC initiative leaders focused on developing a supportive day program for members of the senior center.

Social Capital. The extent to which the initiatives partnered with organizations outside the lead organization also influenced their approach to implementation. For example, initiatives that developed and maintained a multisectoral action team had input and support from a more community-wide network of stakeholders. Through their network, the action team often advanced the development of programs throughout the community, coordination of services across community organizations, and increased outreach to residents in the public at large. For example, one initiative that had an action team comprising members from the local senior center, a residential care center, a dementia-focused non-profit organization, and a continuing care retirement community was able to develop a town-wide dementia-friendly calendar:

*“We created a town-wide calendar for dementia-friendly programming. I was talking with the [assisted living] that also had a caregiver support group during the day. I scheduled ours at night so that if there’s someone who couldn’t come during the day, they could come at night.”*

In addition, initiatives leveraged their social capital through partnerships outside the action team for broader community impact. For example, some initiatives partnered with local age-friendly community initiatives, and in some cases, the DFC initiative was positioned as a subcommittee of the age-friendly work. For example, one DFC initiative leader described partnering with an age-friendly initiative to work collaboratively towards related goals:

*“[Name of Local Hospital] was working on an age-friendly health systems initiative. We were able get in some their meetings so that we can try to better integrate dementia-friendly into age-friendly. I think originally they were seen as separate initiatives, but I think it really is beneficial when they are integrated and working together.”*

On the other hand, some initiatives did not rely, nor focus, on partnerships outside of their organization to accomplish DFC initiative work, especially when they were focused more so on enhancing the dementia-friendliness of their own organization. For example, one initiative developed a memory café at the senior center targeting existing clients. However, the leader reflected on the “low turnout to the café” because “the town is small” and if the memory café “only has two or three people, then no one wants to come”. In an effort to engage more PLWD, the participant shared how they were thinking of partnering with senior centers in neighboring towns to coordinate memory cafés at different locations.

Human Capital. Regardless of the implementation approach, we found it common for DFC initiatives to be led by motivated individuals who were passionate about improving the quality of life of PLWD and their care partners. However, differences in the leaders’ overall vision and decision-making influenced the initiatives’ approach to implementation. For example, reflecting a community-wide approach, one initiative leader with a social work background, whose role within the COA focused on outreach, shared:

*“I just decided what populations in town I wanted to target first. I needed to be out in the community. For my dementia-friendly campaign, I spend my time with the Lions Club, Rotary Club, confirmation classes, Boy Scouts, Girls Scouts. I go to churches and do presentations after the services.”*

This example demonstrates how the approach of this particular leader—to be the “boots-on-the-ground” outreach person in the community to spread awareness about DFC initiative programming—resulted in a community-centered focus on the work. On the other hand, there were also motivated leaders of DFC initiatives who envisioned developing a culture of acceptance and support of PLWD within their organization. For example, one participant described how having an initiative that advocates for the inclusivity of PLWD allows the organization’s members living with dementia to feel more welcome when they attend events or activities at the organization:

*“We had a gentleman living with dementia attend our exercise group [at the senior center]…he had been kicked out of other centers before because he was saying inappropriate things to other people…So, each time a new person came into the group, I [educated them about dementia] and [encouraged them] to have compassion for him and his wife.”*

### 3.2. Case Illustrations

We now present three case examples to further illustrate the above-reported themes (see Supplementary Table S2 for summaries of each case). The first two cases demonstrate the key difference between initiatives focused on the community at large in contrast to initiatives focused more so on the lead organization. The third case describes an initiative that initially focused on the community at large but then transitioned to the work of their own organization because of circumstances related to the COVID-19 pandemic. Throughout our presentation of each case, we highlight financial, social, and human capital as important contexts for the initiative’s implementation approach.

#### 3.2.1. Case #1: Toward a More Dementia-Friendly Town

This initiative began when the COA director became aware of the growing age-friendly movement. As a result, she drew on pre-existing social capital to partner with academic researchers to conduct a needs assessment in the community. The needs assessment received financial support from the municipal government and brought together several community leaders (e.g., librarian, police officer, PLWD) through focus groups and interviews. These key informants ultimately became the members of the age-friendly community (AFC) action team, which included the DFC initiative as a subcommittee of the local AFC initiative that met monthly to discuss progress.

In addition, utilizing the human capital of the COA, this initiative was run by an experienced and motivated senior center director with a personal and professional background in dementia. This background inspired a strong vision of a community-wide emphasis on becoming more inclusive, intentional, and supportive of PLWD. This leader was able to advocate to town officials on behalf of the initiative to raise awareness about the importance of supporting PLWD and their care partners in the community:

*“[The process of signing the DFM pledge] put me in front of the select board for them to sign…It formalized this work group a little bit, and it also put something in front of the town administrator to say, ’Town employees need to be trained, and they need to know this, and the town should go be on the forefront of it, and then the rest of the town can follow.”*

Reflecting ways in which many DFC initiatives that focused on the community at large also engaged in work focused on their own organization, this initiative was able to utilize funding from the municipal government to support the development and sustainability of their memory café:

*“There is a line item in the [COA] budget for $20,000 [to use towards dementia-related programs and services]…I think memory cafés are just a phenomenal way for a caregiver to have an hour and a half to be with other caregivers and in a very comfortable space so that they can share their anxieties, their thoughts, their resources, their everything.”*

#### 3.2.2. Case #2: Toward a More Dementia-Friendly Senior Center

This case provides an example of an initiative whose implementation approach focused more on the lead organization. Drawing on the human capital of the municipal senior center, a part-time staff member led the initiative with support from the COA director, social worker, outreach coordinator, and other staff members. This leader was not paid to designate hours towards the initiative; their time coordinating the DFC initiative effort was provided as part of their role within the COA. The leader of this initiative expressed both their passion for helping PLWD and their care partners who attended the center, as well as the human capital to support this effort:

*“We have eight employees at our senior center…We know the majority of people who have any level of dementia [at the senior center], whether it’s an official diagnosis or not.”*

In addition, unlike other initiatives that developed multisectoral action teams involving a variety of community organizations, the leader of this initiative led discussions on dementia-friendly work at weekly senior center staff meetings. Due in part to the center’s highly regarded reputation in the town (e.g., the participant described a culture of “we ask, they come”), the participant described not needing to rely on outside partnerships to engage PLWD and care partners in their dementia-friendly work. Instead, the DFC initiative drew on the human and tangible capital of the senior center—such as the outreach coordinator, high-quality facility space, and a center newsletter—to reach PLWD and their care partners, who were members of the senior center. 

Furthermore, unlike other initiatives that had more human, financial, and social capital to support dementia-friendly work during the pandemic, the leaders of this initiative were focused on crisis response to COVID-19. As a result, the initiative had planned some dementia-friendly programming for senior center members (e.g., a memory café), but these programs did not happen because of the pandemic. For example, at the time of the interview, the DFC initiative leader was starting to plan training for center staff and volunteer drivers to increase awareness of the signs and symptoms of dementia. The participant reflected that this training would have happened earlier if the pandemic had not occurred.

*“Instead of planning and executing [the DFC initiative work], it was all-hands-on-deck [for crisis response to the COVID-19 pandemic]. We did meals and food delivery the entire time. We just opened our center this week…So we’re just getting back out of crisis mode.”*

#### 3.2.3. Case #3: ‘Living’ at the Senior Center for Now

This case provides an example of an initiative that transitioned from a more community-wide approach to an organizationally focused initiative. The initiative was located within a COA and began with a focus on making the community more dementia-friendly. Drawing on their pre-existing social capital, they assembled an action team that included representatives from multiple sectors, such as the local government, library, hospital, assisted living, chamber of commerce, and a person living with dementia. In addition, they conducted training with community leaders (e.g., first responders and local government officials) and enhanced local services through a memory café that served existing senior center clients while also targeting the community at large (e.g., through efforts to advertise the memory café in the community to make it known that this service was available at the COA). 

The leader of this initiative shared that the benefits of having the multisectoral action team included “increasing the visibility of the initiative” and “allowing for a greater range of relationship-building in the community”. As a result, the participant expressed that, ideally, the initiative should be a more town-wide activity and proposed that initiative might be better suited with the library as its auspice organization. However, as a result of transitions in leadership and the COVID-19 pandemic, the multisectoral action team stopped meeting, and DFC initiative efforts became focused on dementia-friendly programming at the senior center/COA:

*“Our hope was to not have it be a Council on Aging initiative, but to have it be something that was a town activity. We were actually thinking that the library might be a better place for it to live moving forward, but they were having staffing decreases and then COVID happened, and we have more staff, so it’s staying, it’s living here for now.”*

The initiative did not have any outside funding sources, and, as a result, the staff of the COA as well as the action team dedicated time to the initiative in kind. As such, because of the effects of the pandemic and transitions in leadership, it was not possible to continue the community-wide DFC initiative efforts:

*“We were in a transition phase starting in the fall before COVID because the person who took on the chairmanship could not continue…Eventually the visiting nurse and myself decided we would split [the leadership role] until we could identify another volunteer leader of the group, but then COVID happened.”*

## 4. Discussion

To accelerate knowledge development on DFC initiatives, we conducted semi-structured, qualitative interviews with on-the-ground practice leaders affiliated with a network in MA to identify variations in approaches to implementation. Our analysis revealed a key distinction in DFC initiative implementation: the extent to which the primary target of change was the community at large versus the initiative’s lead organization. In addition, our findings indicate how varying degrees of financial, social, and human capital influence the implementation approach. 

Buckner and colleagues (2022) discuss similar themes in reflecting on the results of their multi-site case study of DFC work in England. They state: “There are a wide variety of origins, organizational characteristics, and ways of operating among DFCs in England. While the majority are defined by their geographical location, some are ‘Communities of Interest’ organized around shared identities, interests and places” [31]. In addition, Sun and colleagues, (2022)—the only other study we know of that has used formal research methods to understand the experiences of DFC initiative leaders in the United States—also found that financial capital (e.g., dedicated funding for DFC initiative efforts), social capital (e.g., multisectoral partnerships), and human capital (e.g., leadership and staff time) influenced the scope of DFC work during the COVID-19 pandemic [18].

In addition to finding differences among initiatives, we also found differences in the implementation approach within the same initiative over time. Intra-initiative shifts in focus were especially salient given that our study was conducted during the COVID-19 pandemic. We found in this context that some initiatives shifted their initial community-wide focus to a more organizational focus as a way to sustain the initiative in light of additional stressors from the pandemic. This insight has relevance for the presumably majority of communities that do not have sufficient financial, social, nor human capital—in times of crisis or in the everyday—to develop a DFC initiative for the whole of their community. Such circumstances are especially likely for communities across the United States, wherein there is no federal policy mechanism to systematically direct public funds to age- and dementia-friendly community work [44].

Accordingly, our findings can help to inform strategies to support DFC initiative efforts across communities with varying levels of capacity for dementia-friendly efforts. Our results indicate the potential value of cultivating dementia-friendly change within organizational communities, perhaps providing a foundation for expansion to other organizations and across networks over time. This approach is consistent with theories of community collaboration, such as Asset-Based Development and Strategic Doing™ [40]. Such theories emphasize community-building processes, wherein community change initiatives progressively and incrementally develop the capacity of individuals, organizations, and networks within a community to address multi-faceted, complex social issues over time [38]—starting with the financial and non-financial resources they already have. It also fits with social psychological and developmental theorizing on the importance of achieving goals in building confidence, skills, and motivation toward larger goals over time [45].

Furthermore, our findings suggest the importance of conceptualizing DFC initiatives from theoretical standpoints beyond a social planning model, which has permeated guidance for both DFC and AFC practice in the United States and beyond [44]. Social planning models involve bringing together people from diverse sectors to engage in a multi-phase process of assessment, planning, action, and monitoring toward systems change [46]. While some initiatives in our study described incorporating elements of a social planning model, especially vis à vis a community needs assessment, we found that the extent to which their portfolio of actions was targeting the community at large versus their own organization to be a more cross-cutting dimension of difference in implementation. In fact, there was no evidence from any of the initiatives in our sample that their action plan specifically was controlling their programmatic efforts. In this sense, our results indicate the importance of conceptualizing DFC initiatives from other relevant theoretical perspectives, including ecological systems theory [47], complex systems theory [48], and social network theories [49]. These theories can help to orient DFC initiative leaders to more explicitly identify their target level(s) of systems and community change. Doing so would help frame their initiatives’ key inputs, activities, outputs, and outcomes over a specified period of time, thereby strengthening the development and implementation of their initiatives in terms of budget planning, team-building, partnership development, goal setting, process monitoring, and outcomes evaluation. 

Taken together, our findings can help make sense of inconsistent conceptualizations in the global discourse on what a DFC initiative is [21,50,51]. Our distinction between dementia-friendly “communities” in terms of a place-based community as a whole (e.g., town, city, region) in contrast to an organizationally based community (e.g., senior center) can help make sense of complexity even within the United States. For example, Epps and colleagues (2021) have advanced a body of research and practice on how African American churches can be more dementia-friendly. They have found that characteristics such as quiet rooms, assistance into the building, proper signage, name badges for ministry leaders, and respite or caregiver support resources are important components to make churches more inclusive of PLWD and their care partners [52]. In this case, the faith-based organization can be conceptualized as both an organization and a community, which is fundamentally different from a service delivery organization that provides benefits to one individual at a time (e.g., grocery stores, transportation services, etc.). Senior centers, Villages [53], and voluntary clubs are other examples where organizations both provide services and function as a community. As such, dementia-friendly work at these organizations can be considered both DFC initiatives (an effort to make a community more dementia-friendly) and dementia-capable organizations (an effort to make the service provision of an organization more responsive to PLWD and their care partners).

In addition to implications for theory and practice, findings also can help to advance future research, especially given long-standing calls for more theoretically based studies on DFC efforts [16,28]. Of particular note, results can help to inform the development of quantitative measures to assess differences in the implementation approach of DFC initiatives between and within communities over time. Specifically, our study’s key theoretical insight regarding distinct ecological targets for change (i.e., organizational versus community at large) can contribute to more holistic and meaningful measures of implementation beyond categorical measures of whether or not a community is participating in DFC efforts [54], or engaging in specific activities as part of a DFC initiative [39]. The development of such measures is important for advancing larger, cross-site studies on the development and outcomes of DFC initiatives across diverse socio-spatial settings.

Our study has several limitations. The number of communities joining DFM has increased dramatically over the past two years, and we conducted the interviews during the COVID-19 pandemic. We recognize the unique historical context at which time we collected the participants’ narratives on their practice, likely influencing the themes that emerged through our analysis. In addition, having municipal offices on aging is unique to MA, and the extent to which senior activity centers and other aging services providers are leading DFC initiatives across the United States is not known. Therefore, there might be even greater variation in DFC initiative implementation if we included other types of program leads, such as public health departments, regional planning authorities, and healthcare organizations. Furthermore, the initiatives included in our sample generally were not positioning individuals from historically marginalized racial/ethnic groups as initiative leaders, as per the sociodemographic composition of the people we interviewed. It is essential to continue to advance research and practice on DFC initiatives with considerations of structural racism and other intersectional systems of oppression as a focal point [55].

## 5. Conclusions

This study demonstrates the importance of continuing to use research methods to advance theory for DFC initiatives, especially in ways that are rooted in the practice experiences of local leaders. Indeed, guidance for both age- and dementia-friendly practice historically has emerged from international and national authorities [12,15,39]. By understanding how local leaders are operating in the context of these frameworks through work in their own organizations and communities, we can continue to improve research and practice to strengthen the reach, impact, equity, and sustainability of DFC efforts. Doing so holds great importance for more systematically translating global age- and dementia-friendly aspirations into on-the-ground progress for aging societies of today and the future.

## Figures and Tables

**Table 1:** Demographic characteristics of communities in the sample.

	Mean	Min	Max
Total population size	45,532	3745	185,428
% Population 65+	16%	9%	21%
% Population Non-Hispanic White	80%	55%	98%
% Population Bachelor’s Degree or Higher	49%	19%	84%
Median Household Income	$98,699	$39,432	$145,679

Note. Data derived from the US Census Bureau.

## Data Availability

The data are not publicly available to protect the confidentiality of participants.

## Supplementary Materials

The following supporting information can be downloaded at: https://www.mdpi.com/article/10.3390/geriatrics8020045/s1, Table S1: Three Phases of Iterative Coding Development. Table S2: Summaries of Case Examples 1, 2, and 3. Appendix S1: Overview of the Semi-Structured Interview Guide.
