# Supplementary material for: Variation in Implementing Dementia-Friendly Community Initiatives: Advancing Theory for Social Change
Source: Geriatrics (Basel). 2023 Apr 21;8(2):45. doi: 10.3390/geriatrics8020045 (PMC10138101; doi:10.3390/geriatrics8020045)
Supplement: Supplementary file 1 [file geriatrics-08-00045-s001.zip › geriatrics-2270163-supplementary.docx]

**Supplementary Material**

**Table of Contents**

Supplementary Appendix 1: Overview of the Semi-Structured Interview Guide……..Page 2

Supplementary Table 1: Three Phases of Iterative Coding Development………………Page 6

Supplementary Table 2: Summaries of Case Examples 1, 2, and 3………………………Page 7

**Supplementary Appendix 1**

**Overview of the Semi-Structured Interview Guide**

The interview guide was developed as part of a larger study on the implementation of DFC initiatives in Massachusetts. All interviews included six main segments, as outlined below. Responses to the questions in segments #2, #3, and #4 were of most direct relevance to the current study’s research question. We prepared initiative-specific questions under each segment based on background information about the initiatives based on our review of initiative-specific promotional information, such as newsletters, websites, and event flyers, which were available to the public. Listed below are examples of questions under each segment of the interview.

**Segment #1: Introduction and Background Questions**

- How did the idea of having a dementia-friendly community initiative first come about?
- What do you think motivated people to come together in this way? For example, had people in your community worked together on dementia in the past?
- We see that your community became pledged as part of DFM on [DATE]. Can you explain the timing of that development?
- How would you describe the connection between your community’s dementia-friendly and age-friendly efforts (if relevant)?

**Segment #2: Programmatic Emphasis**

*Note. These questions were developed based on materials from Dementia Friendly America that describe the key objectives of DFC initiatives (DFA, n.d.).*

- Has your DFC engaged in any of the following activities? Explain.
  - Educating the public and key community leaders, such as through trainings on dementia awareness or healthy brain aging.
  - Enhancing local services and supports for PLWD and their care partners, such as by creating easier access, greater variety, better quality, or more coordinated services.
  - Making the physical infrastructure more accommodating of PLWD and their care partners, such as through improving mobility and transportation services, outdoor spaces, and housing.

**Segment #3: Leadership and Action Team**

*Note. These questions were developed based on sensitizing concepts from the literature that focus on age- and dementia-friendly community initiatives as network interventions (Greenfield et al., 2022; Pestine-Stevens & Greenfield, 2022).*

- How is the DFC initiative staffed? Do you have a formal number of hours a week that you are allowed to work on the DFC initiative as part of your role at the senior center?
- What would you say is the purpose of the action team? What do you see as the value added of working together? Are there any examples of how the group has accomplished something that the senior center alone would not have been able to?
- What has been the experience of engaging residents with cognitive impairment or dementia involved as part of the action team?
- What has been your experience of engaging residents from historically marginalized groups such as by race or ethnicity towards dementia-friendly goals?

**Segment #4: Sources of Support**

*Note. We asked these questions as part of our partnership with the state leadership team in Massachusetts to inform them about the supports they provide to local communities (DFM, 2019), as well as our sensitivity to local community initiatives as embedded within broader systems contexts (Chaskin, 2001).*

- We are going to share a list of organizations that aim to support dementia-friendly communities throughout Massachusetts. Can you reflect on them and ways in which they might have helped your work?

**Segment #5: Pandemic Effects**

*Note. These questions were developed based on historical events occurring at the time of interviews.*

- Do you think having the DFC initiative made any difference in the efforts to respond to crises?
- We have heard from other communities that the pandemic slowed down dementia-friendly community plans and activities. Has there been any ways in which the pandemic has slowed down your work?
- Others have shared with us that in some ways, the pandemic has brought about new opportunities to engage in this work. Are there any examples of how the pandemic has enhanced your efforts?

**Segment #6: Future Goals and Conclusion**

- Can you share with us broadly what you see as your most important action steps moving forward with the DFC initiative?
- Is there anything else that you would like to share about your experiences?

**References**

Chaskin, R. J. (2001). Building community capacity: A definitional framework and case studies from a comprehensive community initiative. *Urban Affairs Review*, *36*(3), 291–323. https://doi.org/10.1177/10780870122184876

DFA. (n.d.). *New Evaluation Guide for DFA Communities*. Dementia Friendly America. Retrieved November 15, 2021, from https://www.dfamerica.org/spotlight/2021/5/26/new-evaluation-guide-for-dfa-communities

DFM. (2019, February 8). *Leadership Team: Dementia Friendly Massachusetts*. https://dfmassachusetts.org/about-us/leadership-team/, https://dfmassachusetts.org/about-us/leadership-team/

Greenfield, E. A., Black, K., Oh, P., & Pestine-Stevens, A. (2022). Theories of community collaboration to advance age-friendly community change. *The Gerontologist*, *62*(1), 36–45. https://doi.org/10.1093/geront/gnab136

Pestine-Stevens, A., & Greenfield, E. A. (2022). Giving, receiving, and doing together: Interorganizational interactions in age-friendly community initiatives. *Journal of Aging & Social Policy*, *34*(2), 218–236. https://doi.org/10.1080/08959420.2021.2024412

**Supplementary Table 1.** Three Phases of Iterative Coding Development

| **Phase 1** | **Phase 2** | **Phase 3** |
| --- | --- | --- |
| Action team^a^  Advocacy on behalf of PLWD^i^  Dementia-friendly frameworks^b^  Communications platforms^d^  Coordinating services^i^  Connection to age-friendly^h^  Formal recognition of DFC^c^  How the DFC is resourced^d^  Involvement of PLWD^e^  Outreach to PLWD^f^  Needs assessment^g^  Partnerships outside the action team^h^  Programmatic offerings^i^  Staffing^d^  Support from government^h^ | a. Action team^3^  b. Dementia-friendly frameworks^5^  c. Formal recognition of DFC^3^  d. How the DFC is resourced^1,2,3^  e. Involvement of PLWD^3^  f. Outreach to PLWD^2^  g. Needs assessment^5^  h. Partnerships outside the action team^3^  i. Programmatic offerings^4,5^ | 1. Human Capital  a. Staffing  2. Tangible Capital  a. Funding  b. Communications platforms  c. Facility space  3. Social Capital  a. Auspice organization  b. Action team (composition and status)  b. Partnerships outside of the action team  c. Involvement of PLWD  4. Creating Programs and Services  a. Program type  b. Targeting the organization  c. Targeting the community  5. Other activities  a. Advocating on behalf of PLWD  b. Developing a needs assessment |

*Note.* Superscripts (letters) next to each code in phase 1 reflect how excerpts within that code were recategorized in phase 2. Numbers next to each code in phase 2 reflect how excerpts within that code were recategorized in phase 3.

| Cases | Summary |
| --- | --- |
| Case #1: Toward a More Dementia-Friendly Town | The auspice organization of this dementia-friendly community (DFC) initiative was a senior center. The senior center director, in partnership with academic researchers, conducted a community-wide, dementia-related needs assessment. A dementia-friendly multisectoral action team was developed as a subcommittee of the municipality’s age-friendly action team, an important element of the community’s social capital. The DFC initiative further drew on human capital of the senior center (e.g., motivated leader with a personal connection to dementia) and financial capital (e.g., funding from the Council on Aging to support their memory café ) with a focus on enhancing the dementia-friendliness of the community at large. |
| Case #2: Toward a More Dementia-Friendly Senior Center | The auspice organization of this DFC initiative was a senior center. The DFC action team consisted of staff members of the senior center who discussed the DFC initiative as part of regular staff meetings. The initiative drew on the human and social capital of the senior center (e.g., staff time provided in-kind; the senior center’s highly regarded reputation in the town) to engage PLWD and their care partners in dementia-friendly work. As a result of this approach to implementation, their dementia-friendly efforts had focus on members of the senior center. |
| Case #3: “Living” at the Senior Center for Now | The auspice organization of this DFC initiative was a senior center. At first, this DFC initiative developed a multisectoral action team. Leveraging the community’s social capital, the action team conducted dementia-focused trainings with key community leaders (e.g., first responders and local government officials). This enabled the DFC initiative to have a community-wide focus to the efforts. However, due in part to a lack of financial capital and the COVID-19 pandemic, the leaders of the initiative later decided to focus their dementia-friendly efforts on members of the senior center. |

**Supplementary Table 2.** Summaries of Case Examples #1, #2, and #3
